# Zein-Based Nanocarriers: Advances in Oral Drug Delivery

**DOI:** 10.3390/pharmaceutics17070944

**Published:** 2025-07-21

**Authors:** Yuxin Liu, Dongyu An, Xiangjian Meng, Shiming Deng, Guijin Liu

**Affiliations:** Key Laboratory of Tropical Biological Resources of Ministry of Education, School of Pharmaceutical Sciences, Hainan University, Haikou 570228, China; 24110817000022@hainanu.edu.cn (Y.L.); 22110710000004@hainanu.edu.cn (D.A.); 24221055000028@hainanu.edu.cn (X.M.); dsm701@hainanu.edu.cn (S.D.)

**Keywords:** zein, nanocarriers, oral delivery, gastrointestinal barriers, protein

## Abstract

Oral administration remains the preferred drug delivery route but faces formidable gastrointestinal barriers, including enzymatic degradation, solubility limitations, and poor epithelial absorption. Zein-based nanocarriers (ZBNs), derived from maize prolamin, provide a transformative platform to address these challenges. This review synthesizes recent advances in ZBNs’ design, highlighting their intrinsic advantages: structural stability across pH gradients, self-assembly versatility, and a surface functionalization capacity. Critically, we detail how engineered ZBNs overcome key barriers, such as enzymatic/chemical protection via hydrophobic encapsulation, the enhanced mucus penetration or adhesion through surface engineering, and improved epithelial transport via ligand conjugation. Applications demonstrate their efficacy in stabilizing labile therapeutics, enhancing the solubility of BCS Class II/IV drugs, enabling pH-responsive release, and significantly boosting oral bioavailability. Remaining challenges in scalability and translational predictability warrant future efforts toward multifunctional systems, bio-interfacial modeling, and continuous manufacturing. This work positions ZBNs as a potential platform for the oral delivery of BCS Class II–IV drugs’ in the biopharmaceutics classification system.

## 1. Introduction

**Figure 1:**
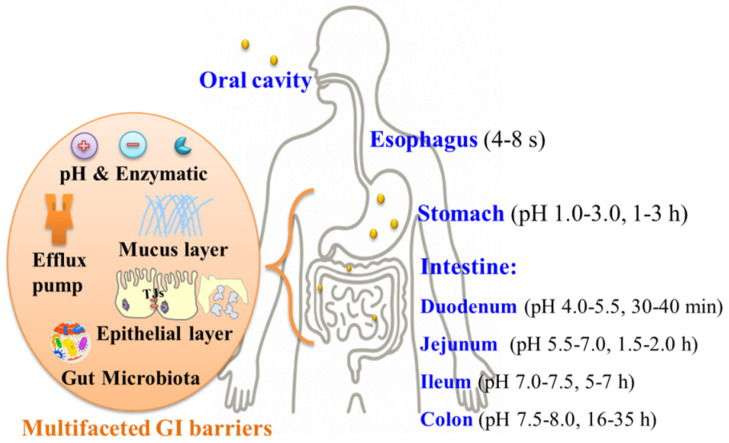
A schematic diagram of the GI sections and their pH, transit time, and multifaceted barriers for oral drug delivery.

Oral administration remains the preferred and most convenient route for drug delivery, constituting approximately 90% of marketed formulations due to its non-invasiveness, its high patient compliance (particularly in chronic disease management), its cost-effectiveness in manufacturing and administration, and the potential for targeted gastrointestinal (GI) therapy, such as the localized treatment of colorectal cancer or inflammatory bowel disease [1,2]. However, achieving effective systemic delivery via the GI tract is significantly challenged by formidable physiological barriers (Figure 1) [3,4,5], including a hostile environment with extreme and variable pH gradients (ranging from pH 1.0 in the stomach to pH 8.0 in the colon) and pervasive enzymatic degradation (e.g., pepsin, trypsin, peptidases that readily cleave susceptible molecules), a viscoelastic mucus layer that physically entraps drugs, and limitations imposed by the intestinal epithelium. These epithelial barriers involve tight junctions (TJs), which severely restrict the paracellular transport of larger or hydrophilic molecules, and efflux pumps (e.g., P-glycoprotein) that actively expel certain drugs back into the lumen. Furthermore, the complex gut microbiota can metabolize therapeutic agents. These sequential and often synergistic barriers pose significant obstacles, especially for poorly water-soluble drugs, low-permeability drugs, and biologics such as peptides, proteins, and nucleic acids, which are highly vulnerable to enzymatic degradation and poor absorption. Consequently, traditional oral formulations frequently fail to achieve adequate systemic drug concentrations, resulting in an insufficient therapeutic efficacy, a highly variable bioavailability, and the potential requirement for higher doses that may increase the toxicity risk, necessitating advanced formulation strategies to overcome these limitations.

In recent decades, nanotechnology has emerged as a transformative strategy to overcome GI barriers. Engineered nanocarriers overcome the significant limitations of oral drug delivery by leveraging their nanoscale properties and design flexibility [6,7]. Specifically, they protect payloads from enzymatic and acidic degradation through the encapsulation within protective matrices or shells. Their small size and tailorable surfaces enhance diffusion through the mucus barrier and interactions with the epithelial layer, thereby improving absorption efficiency. Nanocarriers can further prolong the GI residence time via mucoadhesive coatings or size-triggered retention mechanisms. Moreover, they enable controlled release profiles, either sustained over time or triggered by specific physiological cues (e.g., pH shifts or enzymatic activity), to maximize the payload availability at desired sites of absorption or therapeutic action. Predominant nanocarriers include lipid-based platforms [8], biopolymers (e.g., proteins and polysaccharides) [9,10], synthetic polymeric matrices [11], inorganic nanomaterials [12], and hybrid composites [13]. Among them, protein-based nanocarriers have garnered significant interest due to their exceptional synergy of an inherent biocompatibility and intrinsic multifunctionality [14,15,16]. Composed of naturally occurring proteins, they inherently offer a high biocompatibility, predictable biodegradation into harmless amino acids, and a GRAS (Generally Recognized as Safe) status, which facilitates regulatory approval and enhances patient safety. Crucially, their biological origin grants them a unique functional versatility. Their natural binding domains inherently facilitate targeted delivery via receptor-mediated transcytosis across the epithelium. Their inherent amino acid sequences provide enzyme-cleavable sites that a enable precise, stimulus-responsive payload release triggered by GI proteases. And abundant functional groups (–NH_2_, –COOH, –SH) on the protein backbone permit a facile modification for advanced engineering, such as attaching targeting ligands for enhanced specificity, incorporating stealth polymers to evade immune clearance and penetrate mucus, or crosslinking for improved stability in the GI tract.

Proteins may be derived from animal (e.g., collagen, gelatin, casein, whey, silk, or keratin), plant (e.g., corn alkyd, soy, pea, and lentil proteins), or microbial (e.g., fungal or microalgal proteins) sources [17]. However, many hydrophilic proteins suffer rapid dissolution or extensive swelling in the harsh GI environment, leading to a premature drug release or payload leaching [18]. Furthermore, animal-derived proteins raise concerns regarding potential immunogenicity and contamination risks [19]. In this context, zein (a hydrophobic prolamin derived from maize) stands out as a highly promising material for oral nanocarriers [20,21,22]. Its plant origin ensures a low cost, scalability, an enhanced safety profile, and a GRAS designation [23]. Zein exhibits an exceptional hydrophobicity (~50-fold greater than albumin), intrinsic stability in acidic conditions, and native resistance to gastric proteases, providing upper GI protection [24]. Its unique self-assembly behavior facilitates a simple fabrication into stable nanocarriers [25]. Critically, its surface chemistry readily permits functionalization to engineer desirable properties such as pH-dependent release, mucoadhesion, or enhanced transcytosis [26].

Recently, we provided a foundational overview of the preparation methods, physicochemical characterization techniques, and general pharmaceutical applications of zein-based nanocarriers (ZBNs) [27]. Concurrently, reviews exist focusing on specific applications of ZBNs, including precise cancer therapy [28], tissue engineering [29], controlled drug release [30], and colonic delivery [31]. However, a systematic analysis of their multi-barrier-breaking mechanisms in oral drug delivery remains lacking. This review addresses this critical gap by comprehensively analyzing how the molecular structure of zein governs the self-assembly into functional nanocarriers. Furthermore, it details how these carriers overcome sequential GI barriers: providing enzymatic/chemical protection via hydrophobic encapsulation; enhancing mucus penetration or adhesion through surface engineering; and improving epithelial transport via ligand conjugation. The reviewed applications demonstrate the efficacy of ZBNs in stabilizing labile therapeutics, enhancing the solubility of BCS Class II and IV drugs, enabling a pH-responsive release, and significantly boosting oral bioavailability. Additionally, challenges and future perspectives for the clinical translation of this promising platform are discussed.

## 2. Preparation of Zein-Based Nanocarriers

### 2.1. Characteristics of Zein

Zein, first isolated and named by John Gorham in 1821 [4], is a complex prolamin protein derived from maize. It consists of a heterogeneous mixture of polypeptides primarily aggregated via disulfide bonds, exhibiting variations in the molecular weight, solubility, and charge [32]. The early work by McKinney [33] classified zein into two main fractions: α-zein (soluble in 95% ethanol) and β-zein (soluble in 60% ethanol but insoluble in 95% ethanol). Subsequently, Esen [34,35] proposed a nomenclature system in 1987 and separated zein into three distinct classes: α-zein (75–85% of zein) consists primarily of polypeptides (21–25 KDa) along with a minor component (10 KDa); β-zein (10–15% of zein) features methionine-rich polypeptides (17–18 KDa); and γ-zein (5–10% of zein) is composed of a single type of proline-rich polypeptide (27 KDa). Further studies divided zein into four groups according to its solubility and sequence similarity: α-zein (19 and 22 kDa), β-zein (14 kDa), γ-zein (16 and 27 kDa), and δ-zein (10 kDa) [36]. Notably, these zein fractions may have different molecular weights and proportions when different extraction solvents and methods are used.

**Figure 2:**
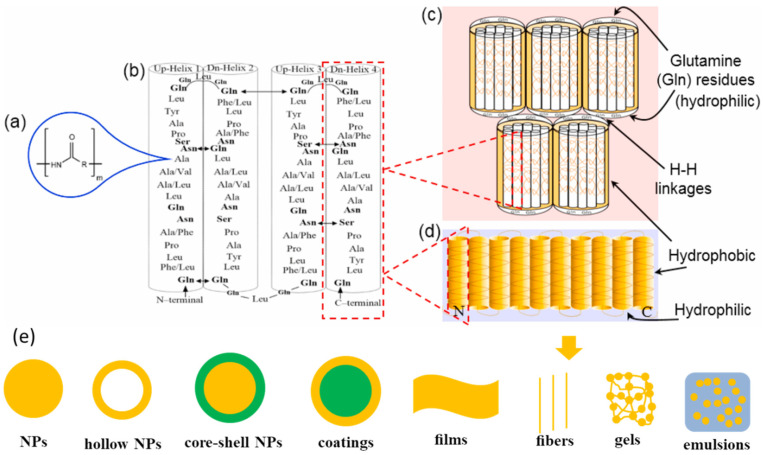
Molecular characteristics of zein: (**a**) basic structure of amino acid; (**b**) hydrogen bonding interactions (↔) between polar groups in adjacent helices; (**c**) proposed model by Argos et al. [41]; (**d**) refined model by Matsushima et al. [42]; and (**e**) schematic presentation of common zein-based carriers. (**a**–**d**) were reprinted with permission from [40], Elsevier, 2021.

A defining molecular characteristic of zein is its amino acid profile. It is exceptionally rich in nonpolar residues (e.g., 19.3% leucine, 9.0% proline, 8.3% alanine) while being deficient in polar acidic and basic amino acids [23]. This unique composition endows zein with strong hydrophobicity and dictates its distinctive solubility behavior, notably being insoluble in water except in the presence of alcohol and high concentrations of urea, alkali (pH ≥ 11), or anionic detergents. Evans and Manley [37,38,39] listed a series of solvents capable of dissolving zein, primarily alcohols and their binary and ternary mixtures. Critically, zein molecules exhibit unique three-dimensional structural characteristics in solutions [40]. A helical wheel model of zein was first proposed by Argos et al. [41], according to the α-helical content and repeat sequence units in methanol (Figure 2a–c). That was a distorted cylindrical bundle of nine adjacent, anti-parallel α-helices. Polar residues on the helical surfaces facilitate intra- and intermolecular hydrogen bonding, enabling planar molecular arrangements. Glutamine-rich turns connecting helices and capping the cylinders further allow the stacking of these planes via side-chain interactions. Refining this model using small-angle X-ray scattering, Matsushima et al. [42] suggested a ribbon-like model for α-zein in 70% ethanol (Figure 2d), which exists as a asymmetric, prism-like particle with an axial ratio of approximately 6:1. The elongated structure arises from the linear stacking of the anti-parallel helices (dimensions of single helix 13 nm × 1.2 nm × 3 nm). There are also some other models (e.g., hairpin [43], coiled-coil triple superhelix [44]).

### 2.2. Engineering Zein-Based Nanocarriers

The structure of zein features hydrophobic lateral surfaces and hydrophilic top/bottom surfaces rich in glutamic acid. This amphiphilic nature facilitates spontaneous aggregation and a subsequent self-assembly into diverse nano- or micro-scale morphologies (Figure 2e), such as micro/nanospheres [25,45], hollow NPs [46], core–shell NPs [47], coatings [48], films [49], fibers [50], gels [51], and emulsions [52]. The performance of ZBNs critically depends on their structural characteristics, such as their particle size, morphology, surface potential, crystal structure, and drug–carrier interactions [28,53]. Optimizing the preparation methodology enables the precise control of these characteristics.

**Figure 3:**
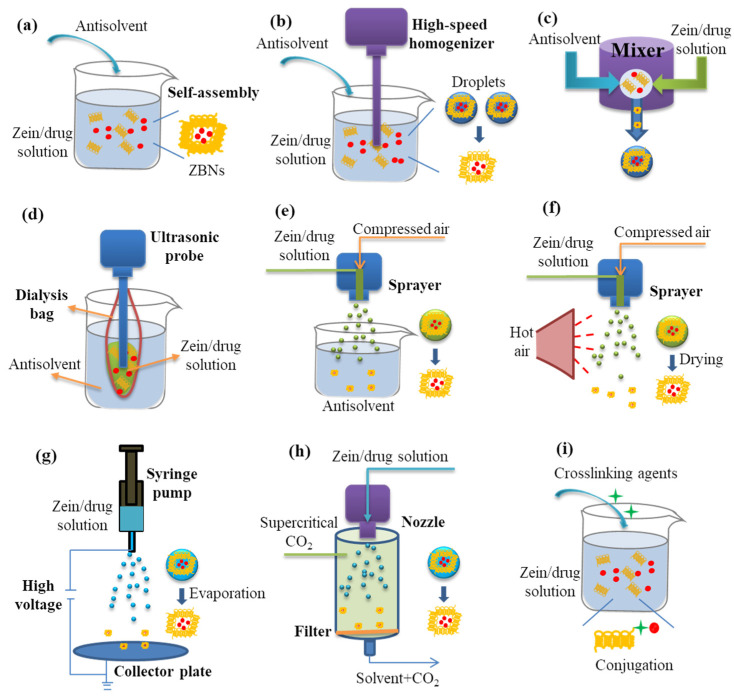
A schematic diagram of the methods for the zein-based nanocarrier preparation: (**a**) phase separation, (**b**) liquid–liquid dispersion, (**c**) flash nanoprecipitation, (**d**) built-in ultrasonic dialysis process, (**e**) atomizing/antisolvent precipitation, (**f**) spray drying, (**g**) electrospraying, (**h**) supercritical antisolvent technology, and (**i**) chemical conjugation.

ZBNs can be prepared via various strategies [27], where common methods are illustrated in Figure 3. Phase separation represents the predominant strategy for fabricating ZBNs, typically using an ethanol–water system [54]. The zein solubility within this system ranges widely (2–60% *w*/*w*) depending on the ethanol concentration. Crucially, the self-assembly of zein molecules is triggered when the ethanol concentration falls below 40% or exceeds 90% (*v*/*v*), first forming coacervates that subsequently solidify into precipitated particles [55]. Conventional phase separation methods, where ZBNs form within the bulk solution, are often plagued by significant particle aggregation, leading to a large particle size and broad polydispersity. To overcome this limitation, several modified approaches have been developed, including liquid–liquid dispersion (shearing the stock solution into small droplets using a high-speed homogenizer) [56], flash nanoprecipitation (rapidly mixing in a multi-inlet vortex mixer) [57], built-in ultrasonic dialysis (combining ultrasonic dispersion with dialysis) [58,59], and atomizing/antisolvent precipitation (utilizing atomizing and antisolvent self-assembly) [47]. In addition, phase separation methods often require a tedious process to isolate the formed ZBNs from the solvent. Conversely, spray drying directly yields a dry powder product, making it particularly suitable for the microencapsulation of non-thermosensitive therapeutics [60]. Zein-based fibers can be readily prepared via electrospinning and electrospraying under an applied electric field [61]. For therapeutics exhibiting a poor solubility in ethanol–water systems, the supercritical fluid technology presents a viable alternative by using supercritical CO_2_ as an antisolvent [62,63,64]. Furthermore, the chemical conjugation of hydrophobic or hydrophilic therapeutics onto the side chains of zein using crosslinking agents can prevent premature drug leakage and enable stimuli-responsive release profiles [65].

## 3. Zein-Based Nanocarriers Overcoming GI Barriers

**Figure 4:**
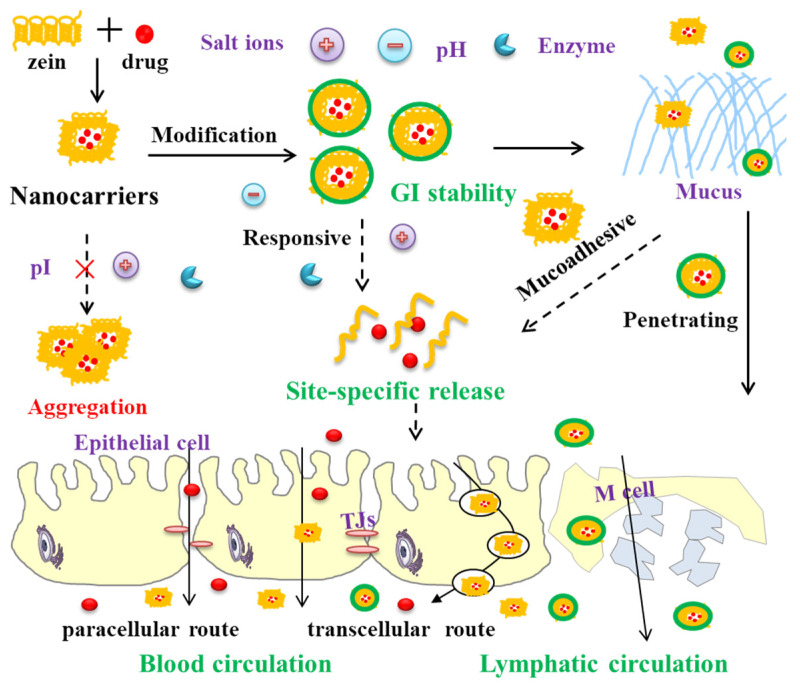
Schematic diagram of zein-based nanocarriers overcoming GI barriers.

The harsh and dynamic GI environment subjects orally administered formulations to sequential barriers: (1) a rapid chemical and enzymatic degradation; (2) entrapment or exclusion by the viscoelastic mucus layer; and (3) limited permeation across the intestinal epithelium. As shown in Figure 4, ZBNs leverage an inherent hydrophobicity and enzymatic resistance to effectively shield payloads from degradation. Surface modifications and formulation innovations mitigate their inherent weaknesses, like intestinal aggregation sensitivity, and actively enhance the mucus penetration and epithelial interaction. This section systematically examines the design principles and efficacy of ZBNs in confronting each of these critical GI barriers.

### 3.1. Stability in GI Tract

The GI tract features a steep pH gradient, from highly acidic gastric conditions (pH 1.0–3.0) to nearly neutral/alkaline intestinal environments (pH 6.5–7.4), coupled with abundant proteolytic enzymes (e.g., pepsin in stomach, trypsin and chymotrypsin in intestines). These factors readily degrade labile payloads (e.g., peptides, nucleic acids) or accelerate the chemical degradation of small molecules, severely limiting their oral efficacy [66,67,68].

ZBNs offer significant protection against the harsh GI environment. Their inherent hydrophobicity, aqueous insolubility at a pH < 11, and resistance to enzymatic hydrolysis effectively shield encapsulated drugs from chemical and enzymatic degradation [69,70]. This protective capacity allows them to transport labile therapeutics significantly further down the GI tract compared to unprotected formulations. However, a critical limitation arises from the protein nature of zein, particularly its neutral isoelectric point (pI ~6.2) [71,72]. As ZBNs enter the neutral or slightly alkaline small intestine, the net surface charge collapses towards neutrality. This loss of electrostatic repulsion is further exacerbated by physiological salt ions, triggering the rapid aggregation and precipitation of ZBNs [40,73]. Consequently, this aggregation compromises the nanoscale size essential for the mucosal interaction and cellular uptake, drastically reducing the dispersion stability and absorption potential in the intestinal tract.

To address the aggregation issues, surface modifications and composite formulations are essential. The covalent conjugation of hydrophilic polymers or charged moieties to side-chain groups of zein weakens its hydrophobicity, shifts its pI, or introduces steric/electrostatic barriers. For examples, Sabra et al. [74] synthesized an amphiphilic zein–lactoferrin (Lf) co-polymer via a carbodiimide coupling reaction. The hydrophilic Lf corona provided steric stabilization and electrostatic repulsion (zeta potential +41.4 mV), which drastically improved the colloidal stability and dispersibility in physiological environments. Ballegooie et al. [75] explored the polyethylene glycol (PEG) modification of zein NPs. The hydrophilic PEG chains create a steric barrier on the NPs’ surface, which minimizes inter-particle interactions driven by hydrophobic effects and van der Waals forces, successfully solving the aggregation issues. These PEGylated zein NPs maintained their size and dispersity for 72 h in DMEM (37 ℃) and 3 months in water (4 ℃).

**Figure 5:**
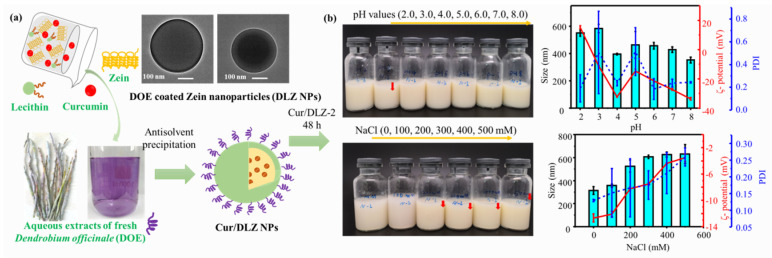
DOE-coated zein NPs as oral delivery vehicles for curcumin (Cur): (**a**) schematic diagram of Cur/DLZ NPs formation; (**b**) the appearance, size, PDI and zeta-potential changes in Cur/DLZ-2 dispersions after 48 h storage at various pH and NaCl concentrations. The red arrow indicates the appearance of precipitate. Reproduced with permission from [83], Elsevier, 2024.

Besides the chemical modification, non-covalent complexation or coating with other polymers (e.g., phospholipids [76,77], polysaccharides [78,79], proteins [80,81]) is widely employed. These polymers form stabilizing shells via electrostatic, hydrogen bonding and hydrophobic interactions. For example, Mariano et al. [82] fabricated core–shell zein/pectin NPs using an antisolvent precipitation/electrostatic deposition method. The high-methoxy pectin coating conferred colloidal stability through electrostatic repulsion (zeta potential shift from +23.2 mV to -22.6 mV) and steric hindrance. This dual-stabilization mechanism enabled the NPs to maintain stability across pH 2.0–8.0, withstand thermal degradation up to 90 °C, and resist aggregation at NaCl concentrations ≤ 50 mM. In our recent work [83], aqueous extracts of fresh *Dendrobium officinale* (DOE) were used as a coating material via an antisolvent precipitation method (Figure 5a). The DOE coating significantly enhanced the stability and dispersity of zein NPs. At an optimal DOE content (~14.24%), spherical DOE-coated zein NPs with small particle sizes (~200 nm) were formed, which exhibited an excellent pH and ionic stability (Figure 5b). Through these synergistic approaches, ZBNs can achieve good stability throughout the GI tract, ensuring sufficient protection for encapsulated drugs and facilitating their controlled release and absorption at the target site.

### 3.2. Mucoadhesion/Mucus Penetration

The mucus layer, a dynamic viscoelastic hydrogel lining mucosal surfaces, significantly impedes oral drug absorption through its complex physicochemical properties and rapid renewal [84,85,86]. Its dense mesh-like network, formed by crosslinked glycoprotein fibers, creates nanoscale pores (20–200 nm) that sterically exclude larger particles. Negatively charged glycosylated residues (e.g., sialic acid, sulfates) and hydrophobic domains within the mucin fibers strongly absorb particles possessing an opposite charge or hydrophobicity, trapping them within the gel matrix. Its shear-thinning behavior, mediated by disulfide bonds and hydrophobic interactions, immobilizes particles under low-shear conditions, hindering the diffusion towards the epithelium. Critically, mucus undergoes continuous turnover and enzymatic degradation, mechanically clearing trapped particles and dissolved drugs before significant absorption can occur.

ZBNs offer promising strategies to overcome the mucus barrier for oral drug delivery. The inherent characteristics of zein enable robust mucoadhesion through hydrogen bonding (amide groups with mucin glycans), hydrophobic interactions (via nonpolar amino acids like leucine), and electrostatic attraction (from partial protonation at GI pH) [87]. This reversible binding to mucus surfaces significantly resists clearance, extending the retention time to prolong the drug release. In the study of Surendranath et al. [88], zein was thiolated via the EDC-mediated conjugation with cysteine, introducing sulfhydryl groups onto the polymer chains. This modification enabled covalent disulfide bond formation between the thiolated zein and cysteine-rich domains of the mucin. The disulfide bonds provided significantly stronger mucoadhesion compared to weaker interactions like hydrogen bonding or chain interpenetration alone. Coating with cationic polymers could further enhance the mucoadhesion via the electrostatic attraction with anionic sialic acid residues in the mucin. Pauluk et al. [89] developed chitosan-coated zein NPs for an oral resveratrol delivery. After coating, the zeta potential shifts from − 20.9 mV (zein NPs) to + 30.5 mV (chitosan/zein NPs) at pH 6.8, resulting in a strong mucoadhesion that was evidenced by a significant increase in the particle size and a reversal of the zeta potential upon incubation with the mucin.

**Figure 6:**
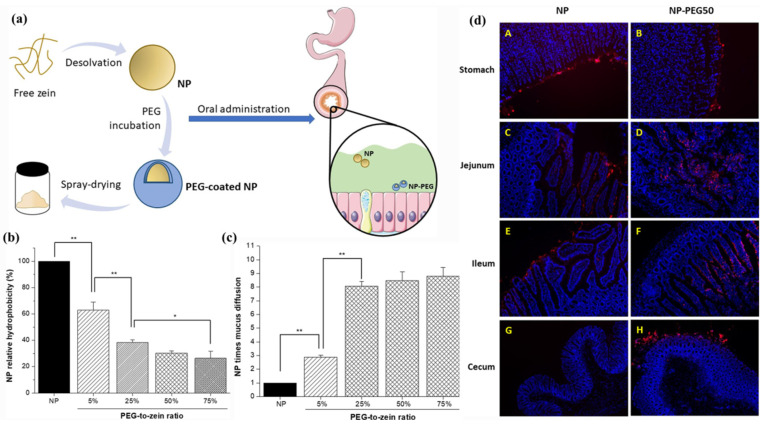
(**a**) Schematic diagram of PEG-coated zein NPs for oral drug delivery purposes. Comparison of (**b**) surface hydrophobicity and (**c**) diffusivity through pig intestinal mucus of NPs with varied PEG-to-zein ratio, *: *p* < 0.05; **: *p* < 0.01. (**d**) Fluorescence microscopic visualization of bare NPs and NPs coated with PEG at PEG-to-zein ratio of 50% (NP-PEG50) in slices of different portions of GI tract of animals, A and B show slices from the stomachs of animals, C and D from jejunums, E and F from ileums, and G and H from cecums. Reproduced with permission from [90], Elsevier, 2021.

Conversely, mucus-penetrating nanocarriers enable a rapid traversal of the mucus layer to reach the intestinal epithelial surface. Surface engineering could shield cationic/hydrophobic moieties of zein, reducing the electrostatic/hydrophobic binding to the mucin, which is crucial to facilitate the penetration of ZBNs through the viscoelastic mucus mesh. For example, Reboredo et al. [90] employed a PEG coating to improve the mucus-penetrating properties of zein NPs for oral drug delivery (Figure 6a). After the PEG coating, the surface hydrophobicity decreased drastically, reaching four-fold lower levels for NPs coated at a PEG-to-zein ratio of 75% compared to bare NPs (Figure 6b). Multiple-particle tracking in ex vivo pig intestinal mucus revealed an ~8-fold increase in the effective diffusion coefficient for PEG-coated NPs (PEG-to-zein ratio≥ 25%) versus bare NPs (Figure 6c). The in vivo biodistribution in rats further confirmed that PEG-coated NPs effectively penetrated the mucus barrier, reaching the intestinal epithelium surface and even the crypts. Notably, only PEG-coated NPs (NP-PEG50) were detected in the cecum, demonstrating enhanced longitudinal transit along the GI tract (Figure 6d). Similarly, Inchaurraga et al. [91] coated zein NPs with a hydrophilic Gantrez^®^ AN–thiamine (GT) conjugate. The GT coating formed a hydrophilic corona (thickness ~15–20 nm) around zein NPs via hydrophobic interactions. This “slippery” surface minimized the hydrophobic entrapment in the intestinal mucus and enabled GT-coated zein NPs (5% GT/zein ratio, GT-NPZ2) to achieve a 28-fold higher effective diffusion coefficient in pig intestinal mucus than uncoated NPs. Biodistribution studies confirmed that GT-NPZ2 reached the intestinal epithelium, while uncoated NPs remained trapped in the mucus layer.

### 3.3. Paracellular/Transcellular Ttransport

The intestinal epithelial barrier, formed by a continuous monolayer of enterocytes interconnected by TJs, imposes formidable biological constraints on oral drug absorption [5,6,92]. Its semi-permeable TJs create size-restrictive paracellular channels (<1 nm pore radius) effectively excluding molecules exceeding ∼700 Da. Simultaneously, the transcellular route is hindered by the hydrophobic lipid bilayer of enterocyte apical membranes, which intrinsically limits the passive diffusion of hydrophilic compounds. Critically, membrane-embedded efflux transporters like P-glycoprotein actively eject substrates back into the lumen. Additional intracellular barriers include the limited endocytic uptake of macromolecules, inefficient endosomal escape, and inadequate basolateral exocytosis [93]. These multifactorial resistance mechanisms drastically reduce the bioavailability of hydrophilic drugs, therapeutic proteins, and nucleic acids, rendering many promising therapeutics ineffective via oral administration.

**Figure 7:**
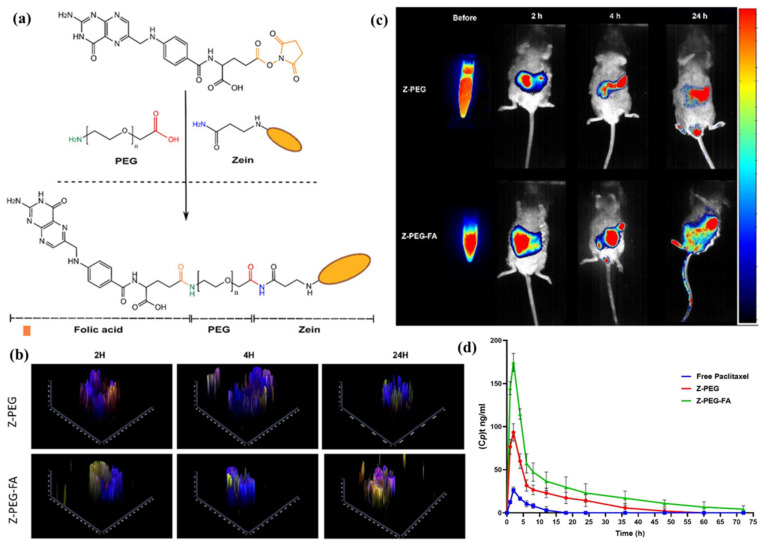
(**a**) The design of folic acid-tethered zein with a PEG spacer (Z-PEG-FA). (**b**) The 2.5 D z-stack for evaluating the green fluorescence intensity in intestinal organoids treated with nanoformulations encapsulating coumarin 6. (**c**) In vivo fluorescence images of rats after the oral administration of DiR-loaded nanoformulations. (**d**) The plasma concentration (Cp) versus time curves of orally administered free paclitaxel and the paclitaxel-loaded nanoformulations. Reproduced with permission from [95], Elsevier, 2025.

ZBNs significantly improve drug absorption by directly enhancing interaction and uptake mechanisms across the intestinal epithelium. Their hydrophobic character promotes strong, favorable interactions with the lipid-rich membranes of the enterocyte. This biocompatible adhesion enables the efficient transcellular transport of the encapsulated payload via multiple uptake pathways [94]. Surface modifications with various ligands can further increase receptor-mediated endocytosis, significantly improving zein NPs’ uptake and transcytosis across the intestinal epithelium. For example, Somaida et al. [95] conjugated zein with folic acid (FA) via a PEG spacer (Z-PEG-FA) (Figure 7a). PEGylation aids the mucus penetration and stability, while the FA receptor-mediated endocytosis enhances the enterocyte uptake for oral paclitaxel (PTX) delivery. An in vitro intestinal organoids study demonstrated a significantly higher accumulation and deeper penetration of fluorescently labeled Z-PEG-FA NPs compared to non-targeted Z-PEG NPs over 24 h (Figure 7b). The in vivo biodistribution in rats showed a prolonged GI retention of DiR-loaded Z-PEG-FA NPs, with fluorescence signals nine-fold, four-fold, and two-fold higher than Z-PEG at 2 h, 4 h, and 24 h post-oral administration, respectively (Figure 7c). Crucially, pharmacokinetic results in rabbits revealed that oral Z-PEG-FA/PTX achieved 7.6-fold and 4.3-fold increases in bioavailability compared to the free PTX and non-targeted Z-PEG/PTX, respectively (Figure 7d). The study of Xing et al. [96] demonstrated that the glucose conjugation to zein (GZ-NPs) significantly enhanced the oral delivery of docetaxel through glucose transporter-mediated transcellular transport and enhanced endocytosis via clathrin, caveolin, and micropinocytosis pathways. Consequently, the glucose modification improved the oral bioavailability from 43.82% to 96.04%, prolonged the mean residence time, increased tumor targeting, and raised the tumor growth inhibition from 77.34% to 89.81%. The surface engineering of zein can further enhance paracellular transport by reversibly opening TJs [97], target activated macrophages [98], and achieve microfold (M)-cell-targeted oral delivery [99]. M-cell targeting allows the carriers and their cargo direct access to the systemic circulation via the lymphatic system, thereby bypassing the hepatic first-pass metabolism [100,101]. By exploiting these versatile mechanisms, ZBNs overcome the absorptive limitations of the intestinal epithelial barrier effectively.

## 4. Applications of Zein-Based Nanocarriers in Oral Drug Delivery

ZBNs exhibit versatile advantages for oral drug delivery by overcoming key GI barriers. Table 1 summarizes 15 representative application cases. Through a material modification, surface functionalization, and composite formulation, ZBNs can be transformed into versatile and sophisticated oral delivery platforms. This section explores how ZBNs tackle critical challenges: drug instability in the harsh GI environment, poor solubility limiting bioavailability, inadequate release profiles, and restricted mucosal/epithelial absorption.

### 4.1. Improving Drug Stability

Labile therapeutics, including peptides, proteins, nucleic acids, and some small molecules, face significant chemical (e.g., hydrolysis, oxidation) and enzymatic degradation within the harsh GI environment. This extensive degradation drastically reduces the amount of the intact drug available for absorption, severely compromising the bioavailability and therapeutic efficacy. ZBNs present a promising solution by exploiting their GI stability and providing encapsulation-mediated protection, thereby improving the drug stability effectively. For example, Fu et al. [54] encapsulated Panax notoginseng saponins (PNSs) within lecithin/zein hybrid nanoparticles (PLZ-NPs). The encapsulation significantly improved the PNS stability in simulated gastric fluid (SGF, pH 1.2 + pepsin, 2h), showing 1.41-fold (R_1_), 1.06-fold (Rg_1_), and 1.34-fold (Rb_1_) higher residual drug levels versus free PNSs. The protection was also pronounced in simulated intestinal fluid (SIF, pH 6.8 + trypsin, 4 h), with stability increases of 1.18-fold (R_1_), 1.34-fold (Rg_1_), and 1.15-fold (Rb_1_). This enhancement of the key saponins is mainly attributed to the enzymatic resistance and barrier function of lecithin/zein NPs. Zhang et al. [102] engineered zein NPs with hydroxypropyl-β-cyclodextrin (ZHC) to protect the curcumin during digestion. The in vitro GI analysis showed that ZHC maintained gastric integrity, limiting the early drug release (16–26% at 60 min) while enabling a sustained intestinal release (56–62% at 180 min). Fluorescence spectroscopy confirmed the curcumin encapsulation within ZHC during gastric transit, and confocal microscopy revealed a digestive enzyme, “protein corona”, formed on ZHC, slowing the release and enhancing stability.

### 4.2. Enhancing Drug Solubility

The poor aqueous solubility of bioactive compounds represents a major challenge in drug development. Over 40% of marketed drugs and 70% of pipeline candidates exhibit this limitation, falling into the biopharmaceutics classification system (BCS) Class II or IV, which significantly restricts oral bioavailability [109]. ZBNs offer a highly promising strategy to overcome this hurdle [30]. The inherent hydrophobicity of zein facilitates strong interactions with poorly soluble drugs, enabling efficient encapsulation during particle formation. The resulting particles (typically 100–250 nm in size) dramatically increase the specific surface area of drugs, enhancing dissolution kinetics. Furthermore, ZBNs act as effective “molecular confinement matrices” by stabilizing encapsulated drugs in a high-energy amorphous state, which inherently exhibits a higher solubility than its crystalline counterpart. For example, quercetin was well encapsulated into the hydrophobic zein core (encapsulation rate 89.41%) via hydrogen bonding and hydrophobic interactions, converting it from a crystalline to an amorphous state [103]. The quercetin-loaded zein core was stabilized by a hydrophilic tea saponin shell formed through electrostatic interactions. This core–shell structure exhibited a 30.16-fold increase in water solubility (from 1.59 μg/mL to 49.54 μg/mL) and a 3.21-fold increase in bioavailability compared to free quercetin. Similarly, ZBNs have been used to improve the solubility of lutein (from 2.67 μg/mL to 215.62 μg/mL) [110], curcumin (from 1.44 μg/mL to ~30 μg/mL) [111], 7,8-dihydroxyflavone (7.12 μg/mL to 231.60 μg/mL) [80], and others.

### 4.3. Controlling Drug Release

**Figure 8:**
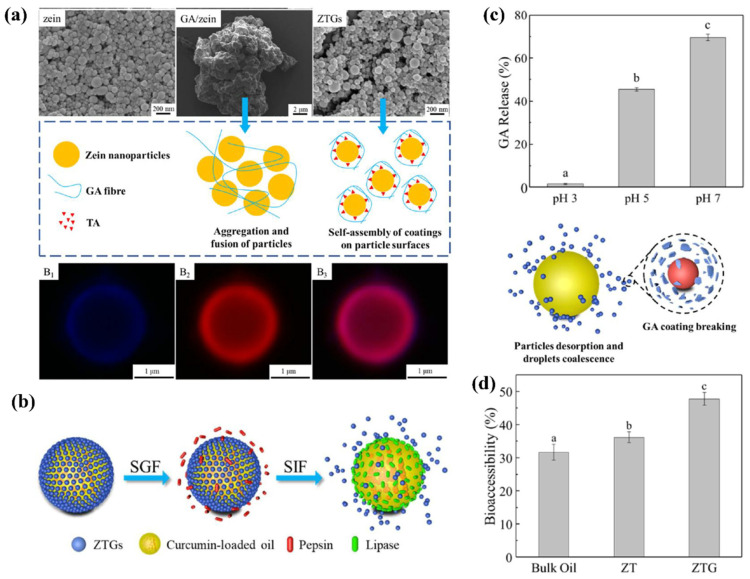
pH-sensitive Pickering emulsions stabilized by zein NPs coated with GA for oral curcumin delivery: (**a**) SEM images and CLSM images of ZTGs (B1: blue fluorescent of GA, B2: red fluorescent of zein particles, B3: overlay fluorescent images of ZTGs); (**b**) schematic diagram of ZTG-stabilized emulsions responsive to GI tract; (**c**) pH-triggered GA release from ZTGs; (**d**) bioaccessibility of curcumin in different emulsions. Different letters (a, b, c) indicate significant differences (*p* < 0.05). Reproduced with permission from [106], American Chemical Society, 2023.

Oral controlled drug release is essential to address challenges such as rapid gastric degradation, toxic or ineffective drug levels due to plasma concentration fluctuations, the poor bioavailability of sensitive drugs, and the non-compliance associated with frequent dosing [112]. ZBNs offer controlled drug release in the GI tract by leveraging the GI stability and protection, pH-triggered release initiation and modulation, and sustained release mechanism combining diffusion control with erosion [113,114]. Surface functionalization further extends their inherent capability, enabling the fine-tuning of the pH response for colon targeting, the enhancement of mucoadhesion for prolonged GI residence, or the incorporation of additional stimuli-responsive triggers [104,105,115]. For example, Li et al. [106] functionalized zein NPs with glycyrrhizic acid (GA) and tannic acid (TA), forming pH-responsive complexes (ZTGs) with a layer-by-layer structure (Figure 8a,b). ZTGs stabilized Pickering emulsions that remained intact under gastric conditions (pH 3) but underwent controlled demulsification over 4 h in intestinal environments (pH 7). This response originated from the GA shedding due to carboxyl group deprotonation above pH 5 (Figure 8c). The pH-triggered demulsification facilitated lipase/bile salt access, increasing the curcumin bioaccessibility to 47.8% versus 36.2% of GA-free emulsions (Figure 8d). In the study by Li et al. [116], zein formed pH-responsive shells on nanocarriers (HEGNs@Z) for the controlled oral delivery of heparin and epigallocatechin-3-gallate (EGCG). The zein shell remained intact in gastric conditions (pH 2.0), which protects its payload, but dissolved rapidly in intestinal fluid (pH 8.0), which triggers drug release. In vitro studies confirmed that HEGNs@Z maintained 84.7% structural integrity in gastric fluid versus only 44% for shell-free HEGNs. Release kinetics revealed significant differential release rates between gastric and intestinal environments, governed by a combination of diffusion and polymer dissolution mechanisms. This pH-triggered mechanism enabled the dual therapeutic effects (anticoagulant and anti-inflammatory) of heparin at the intestinal site for inflammatory bowel disease therapy.

### 4.4. Enhancing Drug Absorption

**Figure 9:**
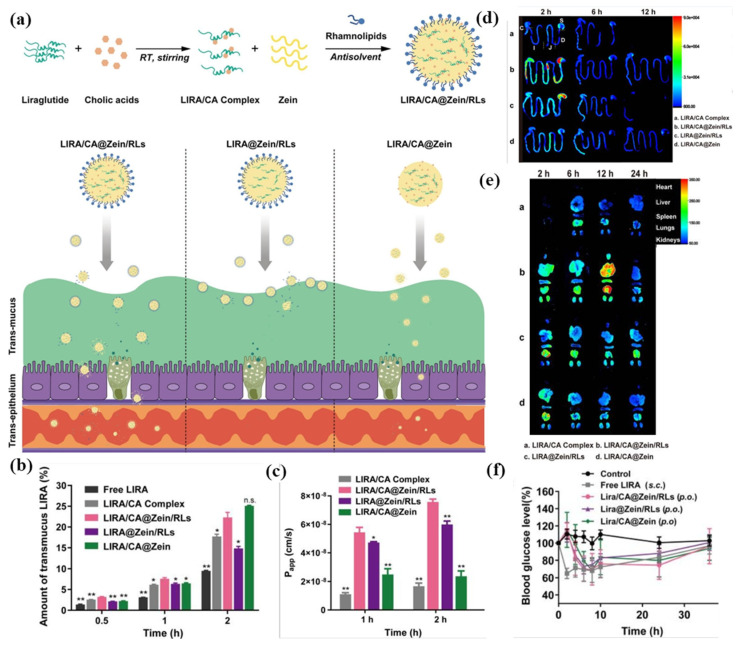
Zein-based nanocomposites (CA@Zein/RLs) accelerating liraglutide (LIRA) oral absorption: (**a**) schematic illustration of preparation and oral absorption of nanocomposites; (**b**) transmucus rate of LIRA across simulated mucus layer; (**c**) transcellular permeability of LIRA in Caco-2 cell monolayers; (**d**) distribution of Cy5-LIRA in GI tract of mice; (**e**) distribution of Cy5-LIRA in main organs of mice; and (**f**) oral hypoglycemic efficacy in type 2 diabetic mice. * *p* < 0.05, ** *p* < 0.01. Reproduced with permission from [107], Springer Nature, 2023.

In addition to GI stability, solubility, and release, oral drug absorption faces major barriers: the GI mucus layer traps and clears particles, while the epithelial barrier limits uptake via TJs and efflux pumps [117]. This is particularly challenging for peptides, proteins, and drugs with low intestinal permeability (BCS Class III/IV). ZBNs significantly boost the oral bioavailability of challenging drugs by improving the mucus permeation and epithelial transport. For example, the oral absorption of liraglutide (LIRA) was significantly enhanced using zein/rhamnolipid (RLs) NPs complexed with cholic acid (CA) (Figure 9a) [107]. Within this delivery system, zein provided enzymatic protection for the peptide, while RLs stabilized the NPs and promoted clathrin/caveolae-mediated endocytosis. Concurrently, the CA complexation reduced the LIRA self-aggregation and accelerated epithelial permeation. This multifunctional synergy resulted in a substantially improved mucus penetration (Figure 9b), enhanced the transcellular transport across Caco-2 monolayers (Figure 9c), enhanced the intestinal permeability (Figure 9d), and improved the absorption into the systemic circuit (Figure 9e). In diabetic mice, the nanocomplex achieved 9.6% oral bioavailability and sustained hypoglycemia (> 24 h) matching the subcutaneous injection efficacy (Figure 9f). In the study by Reboredo et al. [108], zein NPs were coated with PEG to enhance oral insulin absorption in diabetic rats by facilitating penetration through the mucus barrier and access to the epithelium. Compared to uncoated particles, the PEG coating achieved markedly lower minimum blood glucose levels (32% vs. 57.0%), demonstrated a 3-fold higher pharmacological activity (15.0% vs. 4.7%), and yielded a 2.5-fold increase in oral bioavailability (10.2% vs. 4.2%).

### 4.5. Enhancing Oral Bioavailability

The synergistic integration of the stability enhancement, solubility improvement, controlled release, and absorption promotion positions ZBNs as a transformative platform for significantly boosting the oral bioavailability of challenging therapeutics. As shown in Table 2, ZBNs improve the in vivo bioavailability parameters effectively, which enable compounds to achieve systemic exposure levels previously unattainable via conventional oral formulations. For example, Quercetin was encapsulated in zein/2-hydroxypropyl-β-cyclodextrin NPs with an 80.7% encapsulation efficiency (EE) and zero-order release kinetics [118]. In rats, this formulation increased the quercetin relative oral bioavailability to 57% (vs. 4% for free quercetin) by enhancing the intestinal absorption and prolonging the plasma residence time (MRT: 25.4 h vs. 4.9 h). This formulation also reduced endotoxemia severity in mice, with lower TNF-α levels and milder symptoms. Paclitaxel was loaded into zein–sodium caseinate NPs and cloaked with E. coliouter membrane vesicles (OMVs) [119]. The OMVs acted as a protective barrier, delaying the paclitaxel release in gastric conditions and enabling intestinal mucoadhesion. In rats, this biomimetic system increased the paclitaxel oral bioavailability by 7.2-fold (AUC: 2.564 mg·h/L vs. 0.354 mg·h/L) and extended the half-life to 11.7 h (versus 4.3 h for free paclitaxel). Vardenafil was encapsulated in zein–alpha lipoic acid nanospheres via liquid–liquid phase separation, achieving an optimal 147.3 nm particle size and 69.38% EE [120]. The formulation demonstrated a biphasic release: 26.48% at 2 h (surface diffusion) and 59.05% at 24 h (core sustained release). In human trials, it increased the vardenafil bioavailability by 2.5-fold versus commercial tablets (AUC: 198.37 vs. 69.24 ng·h/mL) with a prolonged MRT (11.86 h vs. 5.72 h) and delayed *t_max_* (2 h vs. 1 h).

## 5. Challenges and Future Perspectives

Despite their significant promise for overcoming GI barriers, ZBNs face critical hurdles in clinical translation and commercialization. Key challenges include the following: (1) the inherent zein protein heterogeneity arising from source, extraction, and processing variations, leading to batch-to-batch inconsistencies in the particle size, stability, and drug loading that impede quality control and performance predictability; (2) difficulties in predicting the in vivo fate due to the dynamic GI environment (pH shifts, enzymes, microbiota, mucus turnover), hindering reliable design and bioavailability forecasts; (3) substantial engineering obstacles in scaling up lab-based fabrication to robust, cost-effective, and continuous manufacturing processes compliant with Good Manufacturing Practice (GMP), specifically concerning high solvent costs and removal/toxicity issues, achieving sterilization without compromising particles integrity, ensuring long-term stability during lyophilization and subsequent redispersion, and maintaining batch uniformity for consistent critical quality attributes (CQAs); (4) biocompatibility concerns, particularly regarding the long-term systemic immune response and the necessity for rigorous evaluations of long-term systemic toxicity, requiring studies on organ disposition, the chronic inflammation potential, immunotoxicity, and effects on vital organ systems [132]; and (5) a complex regulatory pathway requiring a comprehensive CQA characterization, extensive safety/toxicology profiling (acute, sub-chronic, chronic, genotoxicity studies incorporating immunogenicity and long-term effects assessment), the clear demonstration of therapeutic advantages over existing platforms, and a robust definition of manufacturing processes [133].

Addressing these challenges necessitates innovative strategies: (1) advancing material science through sophisticated chemical modifications and hybrid system designs to enhance stability, reduce immunogenicity, and improve functionality [134,135]; (2) developing next-generation stimuli-responsive ZBNs triggered by disease-specific enzymes, redox gradients, or microbiota metabolites for precise spatiotemporal control and targeting [136]; (3) bridging the in vitro–in vivo gap utilizing advanced physiological models (e.g., gut-on-a-chip incorporating fluid flow, mucus, and microbiota) [137,138] and AI-driven predictive modeling [139,140]; and (4) accelerating translation by adopting continuous manufacturing platforms, such as microfluidics (offering precise control, high reproducibility, and potential for continuous GMP production) and spray drying (a scalable, established method for stable dry powder production, requiring optimization to prevent aggregation and ensure redispersion), coupled with a proactive regulatory engagement supported by robust preclinical safety data [141,142]. Concerted multidisciplinary efforts are essential to transform ZBNs from promising prototypes into clinical practice, particularly for high-impact applications like localized GI disorders, challenging BCS Class III/IV drugs, and biologics’ delivery.

## 6. Conclusions

ZBNs represent a promising multifunctional platform for oral drug delivery, leveraging their inherent hydrophobicity, pH stability, and protease resistance to provide fundamental payload protection. Engineered fabrication and functionalization strategies equip ZBNs with the capacity to overcome critical GI barriers. Specifically, hydrophobic encapsulation shields labile therapeutics from degradation, while surface modifications (e.g., with ligands or polymers) optimize mucus interactions for enhanced penetration or adhesion. Furthermore, receptor-mediated transcytosis mechanisms (e.g., glucose/folate conjugation) significantly enhance epithelial transport. Studies consistently demonstrate ZBNs’ efficacy in stabilizing labile compounds, improving the solubility of BCS Class II/IV drugs, enabling a controlled release triggered by pH shifts or enzymes, and boosting oral bioavailability. However, realizing the full clinical potential requires addressing key translational challenges, particularly batch-to-batch variability, unpredictable in vivo dynamics, and scalable manufacturing constraints. Consequently, future research must prioritize developing multi-stimuli-responsive systems activated by disease biomarkers, establishing predictive multi-scale models of gut bio-interfaces, and advancing continuous microfluidic manufacturing processes.

## Figures and Tables

**Table 1:** Typical applications of zein-based nanocarriers in oral drug delivery.

Material	Drug	Key Strategy	Key Outcome	Reference
Zein, lecithin, β-sitosterol	Panax notoginseng saponins (PNSs)	Protection from GI degradation; lecithin coating to mimic lipoprotein structure	Improved PNS stability in GI tract; increased intestinal absorption; 1.71 × higher oral bioavailability than free PNS	[54]
Zein, lactoferrin (Lf)	Rapamycin; Wogonin	Active tumor targeting (Lf receptor); synergistic mTOR/PI3K/AKT inhibition	Enhanced cellular uptake and cytotoxicity; superior tumor suppression in breast cancer model; sequential drug release	[74]
Zein, soy lecithin, carboxymethyl chitosan (CMC)	Resveratrol	Ternary complex with CMC coating for stability	2.55 × higher drug dissolution; 2.27 × higher bioaccessibility; 1.69 × higher ABTS^+^ scavenging; > 68% drug retention after 45-day storage	[79]
Zein, pectin	Hesperetin	Pectin shell prevents aggregation in GI tract; zein solubilizes hesperetin for micelle uptake	Improved colloidal stability (pH 2–8, NaCl ≤ 50 mM, 4-week storage); 5.6 × higher bioaccessibility	[82]
Zein, dendrobium officinale extracts (DOE), soy lecithin	Curcumin	Plant extract coating for stability and synergistic bioactivity	Good aqueous stability and redispersibility; 3.0 × higher bioaccessibility; 6.8 × higher ABTS^+^ scavenging; DOE coating enhanced cellular uptake and antitumor activity	[83]
Zein, chitosan	Resveratrol	Chitosan (+) binds mucin (-), prolonging residence; zein controls resveratrol release	Improved gastric protection; enhanced mucoadhesion; sustained resveratrol release	[89]
Zein, PEG, folic acid	Paclitaxel	PEGylation (stability, mucus penetration) and folic acid conjugation (FA-mediated endocytosis/transcytosis)	Enhanced stability in GI fluids; improved uptake in spheroids and intestinal organoids; 7.6 × higher oral bioavailability	[95]
Zein, glucose, soybean lecithin	Docetaxel	Glucose modification targets glucose transporters and enhances endocytosis via multiple pathways	2.19 × increased oral bioavailability; 1.22 × higher cellular uptake; higher tumor distribution and lower systemic toxicity	[96]
Zein, hydroxypropyl-β-cyclodextrin	Curcumin	Cyclodextrin complexation to improve solubility; ionic modulation (Ca^2+^/Na^+^) to control digestion	Sustained drug release and enhanced mucus penetration; Ca^2+^-induced stability increased bioavailability	[102]
Zein, tea saponin	Quercetin	Coating zein core with amphiphilic surfactant to enhance stability and solubility	30.16× increased solubility; enhanced thermal/ionic/pH stability; higher oral bioavailability	[103]
Zein; polydopamine	Indomethacin	Colon targeting via porous structure and polydopamine coating	Enhanced solubility via polymorph modification; sustained and colon-targeted release	[104].
Zein, sodium caseinate	Isoliquiritigenin	Caseinate stabilizes zein NPs; colon-targeted delivery via pH sensitivity	Enhanced cellular uptake in colonic cells/macrophages; prolonged colon retention; reduced ulcerative colitis symptoms in mice	[105]
Zein, glycyrrhizic acid, tannic acid,	Curcumin	pH-responsive glycyrrhizic acid coating for intestine-targeted release	Enhanced stability in gastric fluid; pH-triggered demulsification in intestinal fluid; increased oral bioaccessibility	[106]
Zein, rhamnolipids, cholic acid	Liraglutide	Pre-complexation with cholic acid to reduce self-aggregation; rhamnolipids coating for stabilization and endocytosis promotion.	Protection from enzymatic degradation; enhanced intestinal permeability; sustained hypoglycemic effect > 24 h	[107]
Zein, PEG	Insulin	PEG coating facilitates mucus penetration	3.0 × higher pharmacological activity; 2.5 × higher oral bioavailability	[108]

**Table 2:** Summaries of zein-based nanocarriers for enhancing oral bioavailability in vivo.

Compounds	Carriers	EE (%)	In Vivo Study	Key Bioavailability Parameters ^#^	Reference
Paclitaxel	Folate–zein	87.6	Rabbits	*C_max_* (ng/mL): 21.5→162.21 AUC (ng·h/mL):149.75→1147.25 MRT (h): 5.23→5.88	[95]
Docetaxel	Glucose-modified zein	85	Sprague Dawley (SD) rats	Fr(%): 43.82→96.04; AUC (ng·h/mL): 3686→8078 MRT (h): 17.45→25.15	[96]
Vitamin D3	Zein/sodium alginate	77	Wistar rats	Plasma concentration: ↑3.4-fold Alkaline phosphatase: ↑1.3-fold	[115]
Quercetin	Zein/2-hydroxypropyl- β-cyclodextrin	80.7	Wistar rats	Fr (%): 4→57 *C_max_* (μg/mL): 1.4→3.4 AUC (μg·h/mL): 6.77→94.51 MRT (h): 4.9→25.4	[118]
Paclitaxel	Vesicle-cloaked zein	93	SD rats	*C_max_* (μg/L): 51→132 *t_½_* (h): 4.317→11.708 AUC (mg·h/L): 0.354→2.564 MRT (h): 6.454→17.074	[119]
Vardenafil	Zein/alpha lipoic acid	69.38	Humans	Fr: ↑2.49-fold *t_max_* (h): 1→2 AUC (ng·h/mL): 69.24→198.37 MRT (h): 5.72→11.86	[120]
Curcumin	Zein	98	Wistar rats	Fr: ↑9.17- fold *C_max_* (ng/mL): 186.29→1742.97 AUC (μg·h/mL): 2.21→20.27	[121]
Resveratrol	Zein	87	Humans	*C_max_* (resveratrol): 21.80 ng/mL *C_max_* (metabolite): 986.29 ng/mL	[122]
Cannabidiol	Zein/whey protein	89	SD rats	*C_max_* (μg/mL): 0.232→0.466 AUC (μg·h/mL): 1.657→2.912 MRT (h): 6.654→7.136	[123]
**Quercetin**	zein/alginate–pectin	84.2	SD rats	C_max_(mg/L): 1.33→3.13 AUC (mg·min/L):210.75→606.81 MRT (min): 149.9→229.9	[124]
Gambogenic acid	Zein/phospholipid	76.35	SD rats	*C_max_* (mg/L): 0.21→0.42 *t_½_* (min): 69.32→222.52 AUC (mg·min/L): 50.02→243.49 MRT (min): 192.1→420.95	[125]
Quercetin	Zein/caseinate	82.78	SD rats	Fr: ↑2.34-fold Feces excretion (%): 70→19.4	[126]
Doxorubicin hcl	Zein/hydroxyapatite	44.75	SD rats	*t_½_* (h): 13.91→37.91 AUC (μg·h/mL): 22.47→70.07	[127]
Vitamins	Zein/gum arabic	B6: 61.6 B12: 56.3	Wistar rats	Fr (B6): ↑4.8-fold Fr (B12): ↑2.2-fold	[128]
Atorvastatin	Zein	29.71	Wistar albino rats	*C_max_* (μg/mL): 1.79→8.65 *t_½_* (h): 16.99→20.84 AUC (μg·h/mL): 31.28→117.76 MRT (h): 24.38→27.04	[129]
Astilbin	Zein/chitosan	84.68	SD rats	Fr: ↑18.2- fold *C_max_* (ng/mL): 42→2950 *t_½_* (h): 1.45→2.94 AUC (mg·h/L): 0.29→5.3	[130]
Beta carotene	Zein	68.8	Wistar rats	*C_max_* (μg/mL): 49.21→113.02 *t_½_* (h): 14.35→20.87 AUC (μg·h/mL): 1054.8→2825 MRT (h): 21.37→30.48	[131]

^#^ *C_max_*: peak plasma concentration; AUC: area under the curve; MRT: mean residence time; Fr: relative oral bioavailability; *t_max_*: time to reach plasma concentration; and *t_½_*: half-life of the terminal phase.

## Data Availability

Data are contained within the article.
